# Effect of the Free Radical Initiator on the Production of Castor Oil Maleate Oligomers

**DOI:** 10.1021/acsomega.5c02069

**Published:** 2025-05-01

**Authors:** Dayanne L. H. Maia, Fabiano A. N. Fernandes

**Affiliations:** Departamento de Engenharia Química, Universidade Federal do Ceará, Campus do Pici, Bloco 709, 60440-900 Fortaleza, Ceará, Brazil

## Abstract

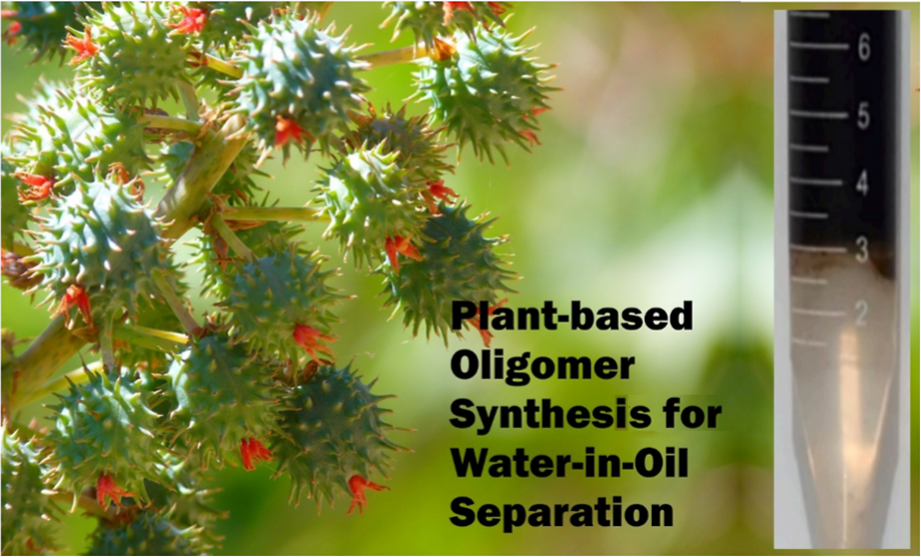

The separation of water from crude oil has long posed a critical challenge in the oil industry, where stable water-in-oil emulsions hinder efficiency and environmental safety. Traditionally, chemical demulsifiers have been employed in this process. However, most of these demulsifiers are petroleum-based, toxic, and environmentally harmful, highlighting the need for sustainable alternatives. Castor oil maleate (COM) oligomers are suitable biobased demulsifiers for water-in-oil separation because they are biodegradable, nontoxic, and environmentally friendly, offering an option toward greener solutions in oil processing. This study synthesized COM oligomers by reacting castor oil and maleic anhydride using six distinct initiators: di-*tert*-butyl peroxide (DTBP), *tert*-butyl peroxy benzoate (TBPB), benzoyl peroxide (BPO), dicumyl peroxide (DCP), potassium persulfate (PSK), and sodium persulfate (PSNa). The findings revealed that DTBP, TBPB, and DCP produced COM oligomers with a higher proportion of longer chain lengths compared to persulfate initiators. COM synthesized using BPO continued polymerizing during storage, increasing the weight-average molecular mass and a higher content of long-chain-length oligomers. In contrast, the other COMs demonstrated typical biodegradation processes, characterized by a reduced weight-average molecular mass and diminished long-chain-length oligomer content. This study highlights the potential of COM oligomers to address longstanding challenges in the oil industry, providing an ecofriendly and efficient alternative to conventional chemical demulsifiers.

## 1 Introduction

Water separation from crude oil has been an old problem in the oil industry.^1^ Stable water-in-oil emulsions are usually formed in underwater–oil reservoirs and during pumping due to shear stress and turbulence. Water in oil can cause corrosion of pipelines and equipment, reduce the efficiency of separation processes, and change the chemical and physical properties of oil-derived products.^2,3^ Thus, the removal of water from oil is essential.

Several mechanical, electrical, and chemical techniques can induce water-in-oil phase separation.^4−6^ Chemical techniques are considered the most economical and effective process. Demulsifiers, hydrate inhibitors, and hydrophobic and dehydrating agents can be applied.

Most demulsifiers for water-in-oil separation are petroleum-based nonionic amphiphilic compounds, which can be toxic and harmful to the environment.^7^ The substitution of these petroleum-based demulsifiers for biodegradable demulsifiers from natural sources has been the focus of many studies.^8−14^

Biobased natural demulsifiers are usually extracted from plants but cannot effectively separate stable water-in-oil emulsions. However, these natural demulsifiers can be chemically modified to improve their effectiveness while maintaining their biodegradability, low toxicity, and environmental friendliness.^10,15,16^

Castor oil maleate (COM) is a suitable biodegradable biobased demulsifier for water-in-oil separation. Recent studies have shown that its applications could efficiently remove water from crude oil using 100 ppm of COM.^15^ Studies have indicated that the efficacy of COM depends on its chemical structure. COM, produced as a mixture of castor oil maleate oligomers containing more than one maleic anhydride in the molecule, is a better emulsifier due to its higher hydrophobicity.^15^

Castor oil maleates are precursors for other industrial products and are used directly as demulsifiers for water-in-oil separation. COM has maleate and carbonate functionalities that can participate in several reactions, including aminolysis and aza-Michael reactions. Epoxidation of COM followed by carbonation with CO_2_ enables the production of a series of polyurethanes.^17^ Diglycidyl ether of bisphenol A can be cross-linked with COM to produce an epoxy system with good coating properties.^18^ COM can also be used in the production of acrylated epoxidized plant-based oil foams, which can be used in packaging materials.^19^

**Figure 1:**
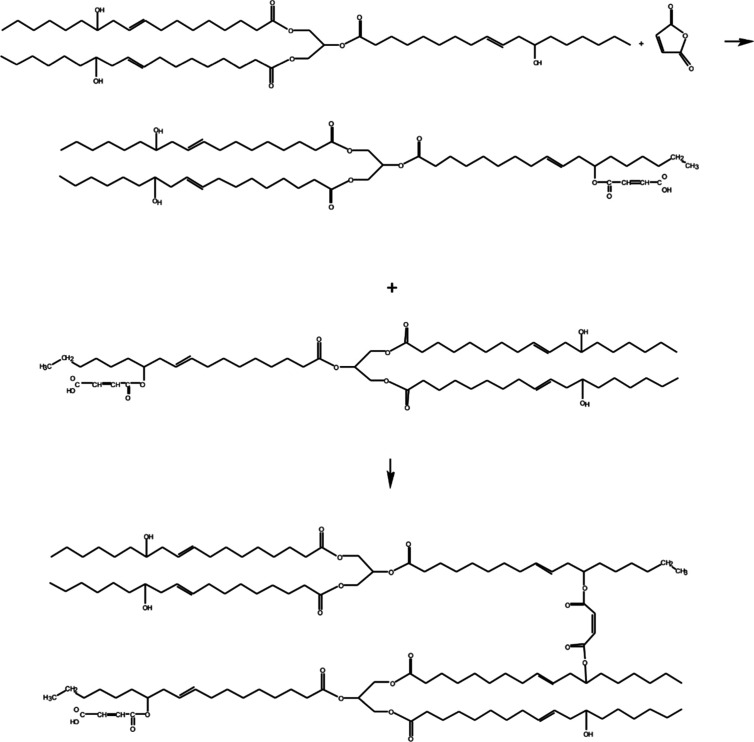
Reaction between castor oil and maleic anhydride by a free radical mechanism.

Production of COM oligomers requires free radical initiators since the autocatalyzed reaction usually results in COM monomers containing a single maleic anhydride in the molecule. Free radical initiators ease the substitution of castor oil hydroxyl groups for maleic anhydride and enable cross-linking of castor oil molecules through a maleic anhydride (Figure 1).

Many free radical initiators are available on the market and could be potential initiators for COM production. In the present work, the castor oil maleate (COM) was synthesized from castor oil and maleic anhydride by using different initiators as a free radical catalyst. Six persulfates and peroxides were evaluated, and the influence of the initiator on the molecular mass distribution was studied. In addition, aged COMs were analyzed to assess the aging process under storage conditions.

## 2 Materials and Methods

### 2.1 Materials

Castor oil was donated by Olveq Indstria e Comércio de Óleos Vegetais (Quixadá, Brazil). Maleic anhydride was obtained from Vetec Qumica Fina (Rio de Janeiro, Brazil). Di-*tert*-butyl peroxide (DTBP, 98%), *tert*-butyl peroxy benzoate (TBPB, 98%), benzoyl peroxide (BPO), dicumyl peroxide (DCP, 98%), potassium persulfate (PSK, ≥99%), sodium persulfate (PSNa, ≥98%), and tetrahydrofuran, inhibitor-free, for HPLC (THF, ≥99.9%) were supplied by Sigma-Aldrich (Darmstadt, Germany).

### 2.2 Synthesis of Castor Oil Maleate

Castor oil oligomerization with maleic anhydride was carried out in a 400 mL stainless-steel batch reactor (Metalquim, Brazil) equipped with a mechanical stirrer and a thermocouple. Castor oil (0.27 mol) and maleic anhydride (0.27 mol) were added to the reactor with a free radical initiator (0.010% wt). The mixture was heated to 140 °C and stirred continuously (600 rpm) for 3 h. These conditions were based on previous works.^15,20−22^ All experiments were carried out in triplicate.

Di-*tert*-butyl peroxide (DTBP), *tert*-butyl peroxy benzoate (TBPB), benzoyl peroxide (BPO), dicumyl peroxide (DCP), potassium persulfate (PSK), and sodium persulfate (PSNa) were used as free radical initiators.

### 2.3 Chemical Characterization

Fourier transform infrared (FTIR) spectroscopy was used to analyze the oligomers’ chemical structure using Cary 630 equipment (Agilent, USA). The spectra were recorded in a scanning range from 4000 to 400 cm^–1^ with a spectral resolution of 1 cm^–1^.

The molecular mass distribution and the dispersity (*D̵* = *M*_w_/*M*_n_) of castor oil and castor oil maleate (COM) were measured by gel permeation chromatography (GPC) using an HPLC system equipped with a Pro Star 355 refractive index (IR) detector and an automated Rheodyne injector using THF as the eluent (flow rate of 1 mL/min). Separation was attained using a TSK Gel G2500HHR column (30 cm × 7.88 mm, 5 μm) maintained at 30 °C. Before injection, the COM samples were dissolved in THF to a 25 mg/mL concentration. The molecular masses were determined based on a calibration curve built using five polystyrene standards with a known molecular mass ranging from 266 to 45,000 g/mol.

The samples were chemically characterized using freshly produced and aged COM. All analyses were carried out in triplicate.

### 2.4 Aging

COM was left standing in closed 50 mL polypropylene tubes for 4 months to determine if aging affected its chemical characteristics. During aging, the samples were stored in the dark at ambient temperature (about 25 to 30 °C).

## 3 Results and Discussion

### 3.1 Synthesis and Characterization of COM

**Figure 2:**
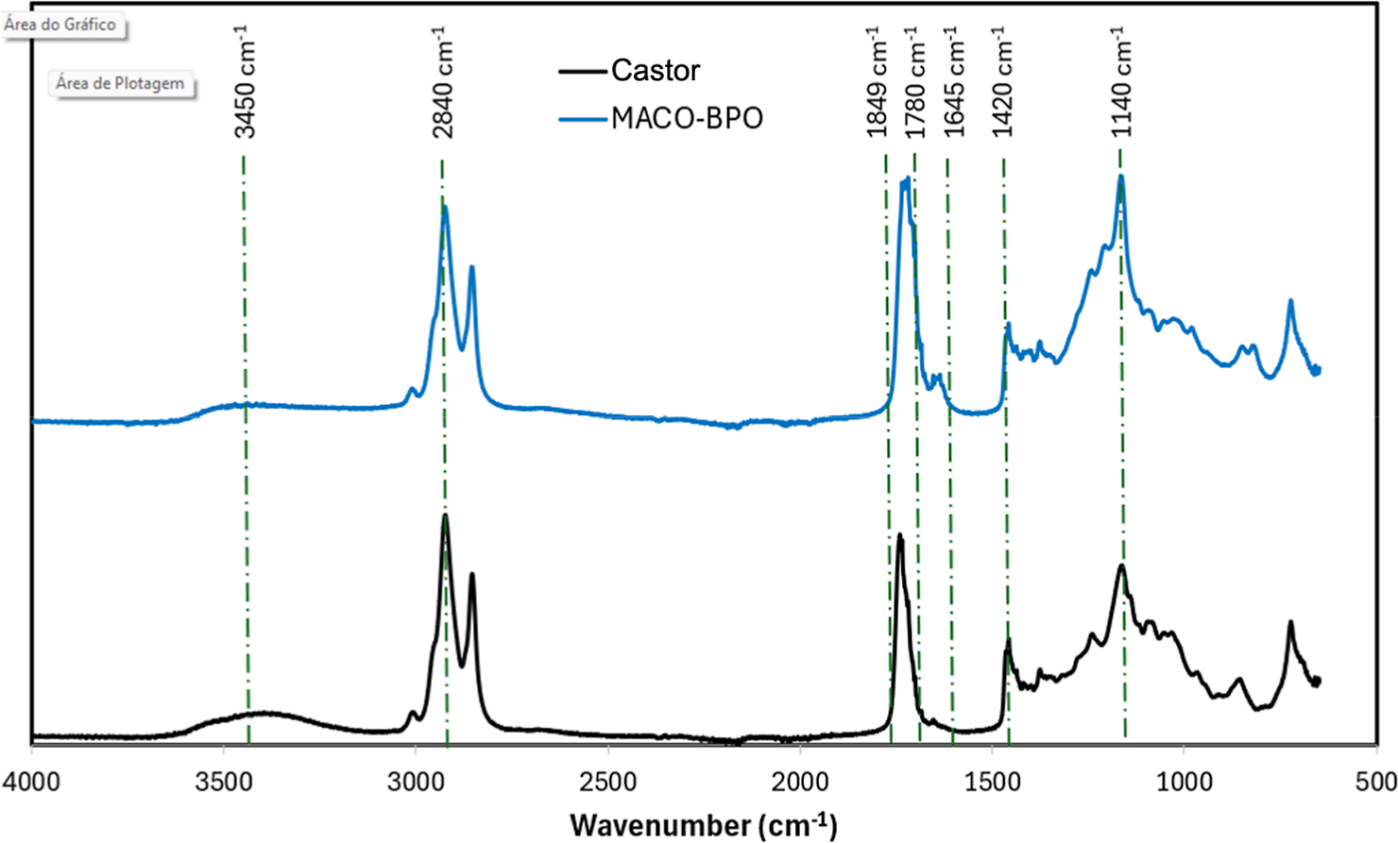
FTIR spectra of castor oil and castor oil maleate.

Castor oil maleate (COM) was synthesized from the reaction of hydroxyl groups in castor oil triglycerides with maleic anhydride at 140 °C using six different free radical initiators. Figure 2 shows the FTIR spectra of castor oil and castor oil maleate. Castor oil maleate is characterized by several bands, including hydroxyl groups at 3450 cm^–1^, alkane stretching at 3000, 2960, and 2840 cm^–1^, carbon–oxygen bond stretching at 1140 and 1780 cm^–1^, alkane bending and wagging at 1420 and 1378 cm^–1^, and carbon double bonds at 1645 cm^–1^. The main differences between castor oil maleate and castor oil are observed at the hydroxyl groups (3450 cm^–1^) and the carbon–oxygen bond of esters at 1140 cm^–1^. The leading characteristic bands of castor oil refer to hydroxyl groups at 3450 cm^–1^ and carbon double bonds at 1645 cm^–1^. The COM spectrum showed a more intense absorption at the carbon double bonds band and a weakened hydroxyl band, indicating successful COM formation. The increase in the absorption at the carbon double bonds is attributed to the rise in the free radical curing sites.^23^ The absence of absorption bands at 1780 and 1849 cm^–1^ (cyclic anhydride) evidences that all maleic anhydride was consumed in the reaction with castor oil.^24^

**Figure 3:**
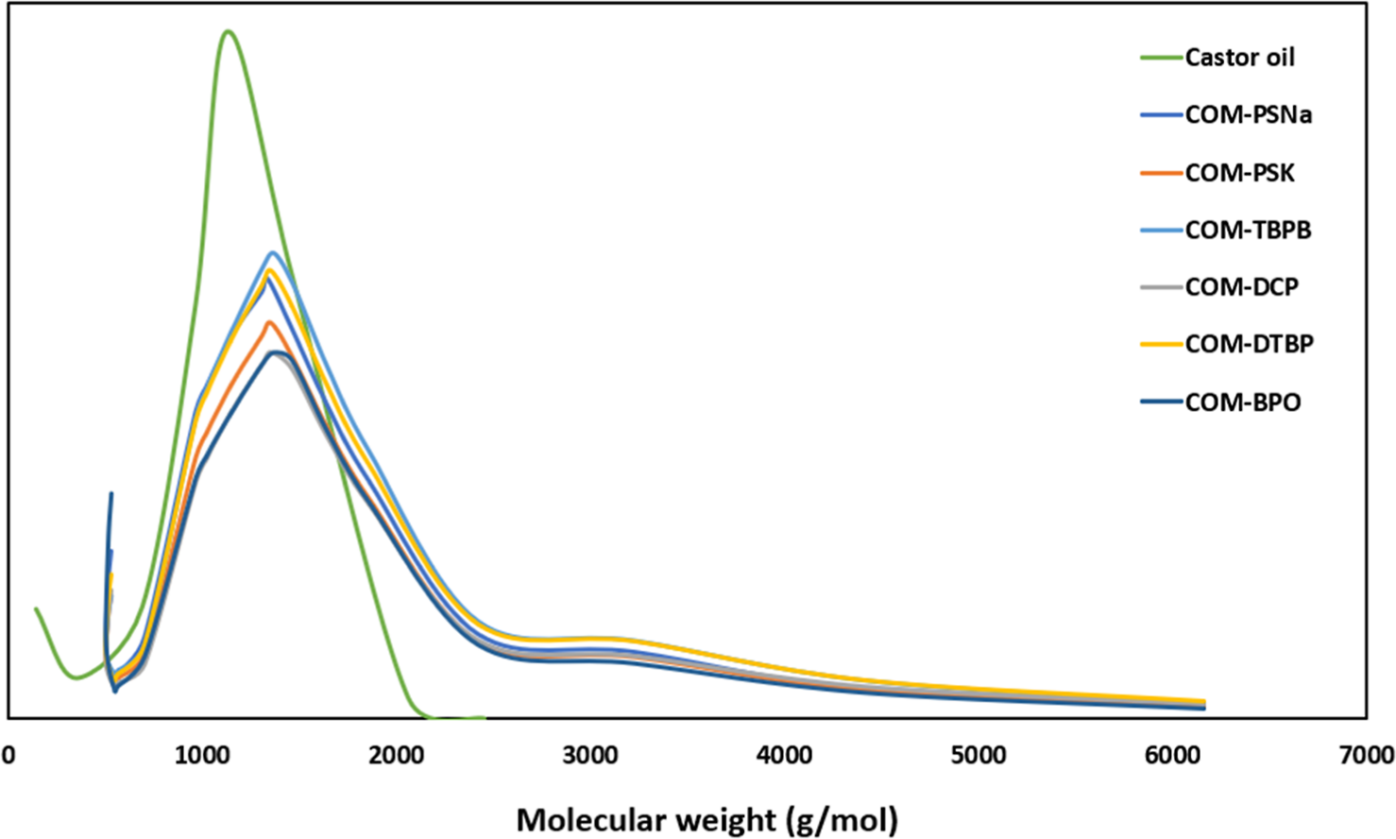
GPC chromatographs of castor oil and castor oil maleates are produced using several free radical initiators.

The molecular mass of all materials was determined by gel chromatography (GPC). Figure 3 presents the typical change in the molecular mass distribution caused by the oligomerization of castor oil with maleic anhydride.

The chromatogram for the castor oil presented a single peak centered at 1129 g/mol, corresponding to the triglyceride molecule. All COM obtained in this study, independent of the initiator, showed a similar molecular mass distribution characterized by narrow dispersity. An analysis of the chromatograms indicates the formation of COM oligomers, mainly dimers and trimers, as previously reported in the literature.^15,20,22,25^

### 3.2 Effect of Initiators

The effects of six initiators on the molecular mass distribution of castor oil maleate (COM) were evaluated. Table 1 presents the number-average molecular mass (*M*_n_), average molecular mass (*M*_w_), and dispersity (*D̵*) of COM. The results indicate that the use of free-radical initiators induces the production of COM monomers and oligomers. Between 52 and 56% of the product will be formed by the COM monomer, and COM dimers, trimers, and oligomers will form the remaining mass fraction. As expected in oligomerization reactions, dimers were higher than trimers and other higher oligomers. Evaluation of the GPC spectra indicated very few oligomers containing more than seven monomers.

**Table 1:** Number-Average Molecular Mass (*M*_n_), Weight-Average Molecular Mass (*M*_w_), Dispersity (*D̵*), and Mass Fraction of Dimers, Trimers, and Other Oligomers of COM Produced with Different Initiators

				oligomers mass fraction (%)				
initiator	*M*_n_ (g/mol)	*M*_w_ (g/mol)	*D̵*	M	2M	3M	4M	5M+
PSNa	1406	1893	1.3	55.3	19.7	15.5	6.7	2.7
PSK	1439	1928	1.3	55.5	19.3	15.4	7.1	2.7
BPO	1401	1899	1.4	56.7	19.4	14.5	6.8	2.6
DTBP	1480	2036	1.4	52.6	19.6	16.1	8.0	3.6
TBPB	1487	2017	1.4	53.6	19.6	15.7	7.8	3.3
DCP	1485	2030	1.4	53.5	19.5	15.5	7.9	3.5

The molecular mass distribution attained with different free radical initiators did not significantly change. The persulfate initiators (PSK and PSNa) produced COM with very narrow dispersity (1.3), while using peroxide initiators slightly increased the dispersity.

The weight-average molecular mass of the oligomers produced using DTBP, TBPB, and DCP indicates that these oligomers have, on average, two maleic anhydrides per triglyceride molecule, while the oligomers produced using PSK and PSNa have only a single maleic anhydride per triglyceride molecule. This result was evidenced by the higher Mw value of the COMs produced by DTBP, TBPB, and DCP than the other COMs at approximately 98 g/mol, the molecular mass of maleic anhydride.

GPC analysis evidenced that the COM comprised mostly monomers and dimers (>71% w/w) and less than 39% w/w of trimers and longer oligomer chains. Using persulfate initiators and BPO resulted in the highest amounts of low molecular mass chains (>75% w/w). DTBP, TBPB, and DCP showed a higher capacity to produce longer oligomer chains.

The half-life of these six initiators increases following PSK < BPO < PSNa < TBPB < DCP < DTBP. Longer oligomers were favored using initiators with higher half-life temperatures (TBPB, DCP, and DTBP). In comparison, initiators with lower half-life temperatures (PSK, BPO, and PSNa) favored the formation of shorter oligomers with narrower molecular mass distribution. Thus, initiators with higher half-life would be preferred for COM production.

As the chain length increases, the hydrophobicity of COM increases, which is ideal for the removal of water from oil emulsions. Dimers and longer oligomers increase the demulsifying efficiency compared to monomers with a similar number of maleic anhydride side branches due to a higher occupation at the water–oil interface. The increase in the molar ratio of maleic anhydride side branches also increases the interaction with the water–oil interface due to a greater interfacial occupation.^15^

However, the viscosity of COM increases when the chain length increases, making the mass transfer of COM into oil emulsions more difficult, as reported for COM–styrene copolymers.^10^ Thus, short-chain-length oligomer mixtures are preferred for water removal applications.

The molecular mass distribution attained herein shows that DTBP, TBPB, and DCP free radical initiators have better efficacy in producing longer oligomer chains. Among these initiators, the costs can be considered a significant factor when deciding which one to employ to synthesize castor oil maleate.

### 3.3 Aging

COMs are biodegradable^21,22^ and tend to decay over time. Aging and storage studies are essential for determining how COMs decay and whether further polymerization occurs during storage.

Table 2 presents the molecular mass distribution of the castor oil maleates aged for four months. The aging process affected the produced COMs differently. The results indicate that aging slightly reduced the number and weight-average molecular mass of COMs, except for the COM produced with BPO. Aging also increased the mass fraction of monomers, with a consequent reduction in dimers and trimers. Between 64 and 67% of the product was formed by the COM monomer, except for the COM produced by BPO. These values are approximately 12% higher than the mass fraction of monomers observed after production. Evaluation of the GPC spectra indicated that most new monomers came from depolymerizing dimers and trimers, while oligomers presented higher stability.

**Table 2:** Number-Average Molecular Mass (*M*_n_), Weight-Average Molecular Mass (*M*_w_), Dispersity (*D̵*), and Mass Fraction of Dimers, Trimers, and Other Oligomers of COM Produced with Different Initiators after Four Months of Storage

				oligomers mass fraction (%)				
initiator	*M*_n_ (g/mol)	*M*_w_ (g/mol)	*D̵*	M	2M	3M	4M	5M+
PSNa	1334	1889	1.3	67.0	14.5	11.5	5.0	2.0
PSK	1361	1910	1.4	67.3	14.2	11.3	5.2	2.0
BPO	1319	1992	1.5	47.9	25.2	15.6	8.1	3.2
DTBP	1393	1967	1.4	64.4	14.8	12.1	6.0	2.7
TBPB	1289	1712	1.3	65.3	14.6	11.7	5.8	2.5
DCP	1408	1989	1.4	65.3	14.8	11.6	5.9	2.6

The COMs produced with persulfate were less affected by aging, and a slight reduction in the levels of *M*_n_ and *M*_w_ was observed. GPC analysis showed that the oligomer distribution shifted significantly toward lower-chain-length oligomers than the newly produced COMs. Mass balance analysis also indicated a probable loss of methyl groups from the castor oil chain. For these castor oil maleates, the percentage of dimers and trimers reduced from 35 to 25% w/w, and the percentage of longer oligomers decreased from 10 to 7% w/w.

The same degradation trend was observed with DCP-, DTBP-, and TBPB-produced COM. GPC analysis showed a significant shift toward short-chain oligomers. Mass balance analysis evidenced the loss of methyl groups from the castor oil chain, resulting in a change in the *M*_n_ and *M*_w_ values. The dimers and trimers reduced from 36 to 27% w/w, and the percentage of longer oligomers from 11 to 8% w/w; a similar percentual reduction was observed for persulfate-produced COM. Such similarity indicates that aging has a similar effect on most COMs.

The reduction in molecular mass and the shift toward short-chain length oligomers were expected since this material was characterized as biodegradable in previous works of our group.^21,22,25^ However, the degradation rate was lower in storage than in landfill conditions, where microorganisms increase the degradation rate of castor oil maleate.

The exception was the aging process of BPO-produced COMs, which presented different aging behavior. Instead of degrading, the BPO-produced castor oil maleate continued polymerizing. The molecular mass distribution shifted toward longer chain-length oligomers. The dimer and trimer content rose from 34% to 40% w/w, and the longer oligomer content rose from 9% to 11% w/w. It is still unclear why BPO-produced COMs continue to polymerize. Still, the phenomena may be related to higher stability and lower autotermination rate of the free radicals formed from BPO.

Continuous polymerization of BPO-produced COMs was verified only under storage conditions, while in landfill conditions, these oligomers have degraded. Although BPO-produced COMs did not degrade under storage, their continued polymerization is not interesting for oil dewatering since the COM tends to increase viscosity and does not quickly diffuse into the oil phase.

## 4 Conclusions

Castor oil maleate (COM) oligomers were successfully synthesized from the reaction of castor oil and maleic anhydride with six different initiators (di-*tert*-butyl peroxide, DTBP; *tert*-butyl peroxy benzoate, TBPB; benzoyl peroxide, BPO; dicumyl peroxide, DCP; potassium persulfate, PSK; and sodium persulfate—PSNa). DTBP, TBPE, and DCP induced the production of COM oligomers with higher content of longer chain lengths, characterized by 4–5% less monomer chains and 22–38% more COMs with 5 or more monomers in the chains, with the weight-average molecular mass ranging between 2017 and 2036 g/mol. The synthesis of longer oligomers was related to the use of free radical initiators with higher half-life temperatures.

The COM produced using the BPO initiator continued to polymerize during storage, increasing its weight-average molecular mass and long chain-length oligomer content and presenting a reduction in monomer chains by 15% and an increase of 23% in chains containing five or more monomers. All other COM showed typical degradation processes of biodegradable materials, leading to a lower weight-average molecular mass and reduction in the content of oligomers with long chain lengths by 7–10%.
